# Development and Validation of an Autophagy-Related Signature for Head and Neck Squamous Cell Carcinoma

**DOI:** 10.1155/2021/1028158

**Published:** 2021-08-11

**Authors:** Chang Liu, Wenling Wu, Meng Xu, Jinglin Mi, Longjiang Xu, Rensheng Wang

**Affiliations:** ^1^Department of Radiation Oncology, The First Affiliated Hospital of Guangxi Medical University, Radiation Oncology Clinical Medical Research Center of Guangxi, Nanning 530021, China; ^2^Department of Medical Oncology, The First Affiliated Hospital of Guangxi Medical University, Nanning, Guangxi Zhuang Autonomous Region 530021, China; ^3^Department of Pathology, The Second Affiliated Hospital of Soochow University, Suzhou, 215000 Jiangsu, China

## Abstract

**Introduction:**

HNSCC is the sixth most frequent type of malignant carcinoma with a low prognosis rate. In addition, autophagy is important in cancer development and progression. The purpose of this study is to investigate the potential significance of ARGs in the diagnosis and treatment of HNSCC.

**Materials and Methods:**

Expression data and clinical information of HNSCC samples were collected from the TCGA database, and a list of ARGs was obtained from the MSigDB. Then, we used R software to perform differential expression analysis and functional enrichment analysis. Further analysis was also performed to find out the survival-related ARGs in HNSCC, and two prognosis-related ARGs, FADD and NKX2-3, were selected to construct a prognosis prediction model. Moreover, some methods were applied to validate the prognosis prediction model. Finally, we used cell lines and clinical tissue samples of HNSCC to analyze the importance of FADD and NKX2-3.

**Results:**

We screened a total of 38 differentially expressed ARGs, and enrichment analysis showed that these genes were mainly involved in autophagy. Then, we selected FADD and NKX2-3 to construct a prognosis model and the risk score calculated by the model was proved to be effective in predicting the survival of HNSCC patients. Additionally, significant differences of the clinicopathological parameters could also be observed in the risk scores and the expression of NKX2-3 and FADD. The expression of FADD and NKX2-3 in cell lines and HNSCC tissue samples also showed the same trends.

**Conclusions:**

ARGs may be a potential biomarker for HNSCC prognosis, and targeted therapies for FADD and NKX2-3 are possible to be a new strategy of HNSCC treatment.

## 1. Introduction

Head and neck squamous cell carcinoma (HNSCC) is the sixth most common malignancy of the world. It arises in the epithelium cells of the upper aerodigestive tract and includes the larynx, paranasal sinuses, oral cavity, nasal cavity, and pharynx [1]. Genetic mutation, environmental exposure, viral infection, and unhealthy lifestyle are the common risk factors for HNSCC [2]. Despite a considerable expansion in our therapeutic repertoire for the management of malignancies in recent decades, mortality from HNSCC has not significantly improved. If diagnosed at the early stage, HNSCC is usually curable [3]. Predicting the prognosis of HNSCC with high accuracy is critical for improving treatment, screening, and surveillance. At present, the TNM (Tumor, Node, Metastasis) stage system is still extensively used as the prognostic indicator to monitor the HNSCC progression. However, it is common to observe significant differences in clinical outcomes among patients at the same TNM stage [4]. Therefore, the identification of novel and reliable prognostic molecular signatures is important for improving the unfavorable prognosis of patients with HNSCC [5, 6]. Additionally, it is significant to develop new therapeutic strategies, so that we could treat tumors with much more precision.

Autophagy is a common biological phenomenon and another name for it is type II programmed cell death. It is a process in order to maintain cellular homeostasis, conducting damaged or defective intracellular components [7]. Previous studies have found cell autophagy is a high conserved process. During this process, cytoplasmic materials are sequestered into double-membrane compartments and subsequently mature into autophagosomes. Then, the cargo is delivered to lysosomes, in which it is for degradation or recycling [8]. Abnormal autophagy function is intimately associated with numerous human diseases, including cancers [9]. It is stated that autophagy could promote tumor development or in some cases could inhibit the occurrence of tumor, by affecting many physiological processes [10]. Autophagy was reported by several studies that it could play a critical role in the formation of HNSCC. Also, abnormal expression of autophagy-related genes (ARGs) is also associated with poor prognosis of patients with HNSCC [10]. This means that ARGs can be used as a novel and reliable indicator of HNSCC patients. However, autophagy is a complex process with several steps. In this process, hundreds of molecular biological changes are involved, and a series of ARGs closely control it [11]. Therefore, compared with a single gene, a model which integrates several ARGs that plays important roles in HNSCC may increase the accuracy of the prediction of the prognosis for patients with HNSCC. These important ARGs could also serve as targets for HNSCC therapy.

To better investigate the influence of ARGs on the survival of clinical patients, we downloaded original gene expression data for HNSCC from the TCGA database. We then used these gene expression data, together with information we obtained from other public databases, to develop a prognostic prediction model as an indicator of overall survival (OS) for HNSCC. We also identified the genes related to clinical outcomes of HNSCC patients. Finally, we used experiments to confirm the results of our analysis, with the purpose of selecting possible targets that can be used for the clinical diagnosis and treatment of HNSCC patients.

## 2. Materials and Methods

### 2.1. Data Acquisition

The Cancer Genome Atlas (TCGA) database is a complete genome-wide gene expression database used for categorizing and detecting genomic abnormalities in a large population worldwide [12]. We downloaded the mRNA expression data and clinical information of HNSCC patients from the TCGA database, including 502 tumor tissues and 44 adjacent nontumor tissues. In addition, a total of 232 ARGs were obtained from the Molecular Signatures Database (MSigDB), by retrieving “Autophagy.” The MSigDB is one of the most widely used and comprehensive databases of gene sets for performing gene set enrichment analysis [13].

### 2.2. Differentially Expressed ARGs and Enrichment Analysis

We analyzed the expression data by using Wilcox test methods through package limma in R [14] to choose the differentially expressed ARGs between tumor and paired nontumor tissues, and the analysis principle was the thresholds of ∣log2 fold change (FC) | >2 and adjusted *p* value < 0.05. Then, we draw heat map, volcano map, and box plot. Gene Ontology (GO) enrichment analysis and Kyoto Encyclopedia of Genes and Genomes (KEGG) pathway analysis were also performed to find the major biological attributes of differential expression ARGs. The visualization of the enrichment maps was performed by R through the “ClusterProfiler” packages [15].

### 2.3. Construction of Prognostic Signature Based on ARGs

Univariate and multivariate Cox regression analyses were applied to screen the survival-associated ARGs in HNSCC (*p* < 0.05, HR > 1). We obtained 2 prognostic ARGs, and then, based on the multivariate Cox regression, we calculated the correlation coefficient of the ARGs. According to the correlation coefficient, the survival-related prediction formulas were performed to build prognostic models. We evaluated the risk score of all HNSCC samples through the prognosis prediction model. To further assess the survival differences, we first divided the HNSCC patients into a high-risk group and a low-risk group, according to the median value of the risk score. Then, Kaplan-Meier (K-M) methods were applied to compare the high-risk and low-risk groups.

### 2.4. Validation of the Prognosis Prediction Model

Clinical data were extracted from HNSCC samples, and risk score was performed on these samples. Univariate and multivariate Cox regression analyses were performed to confirm the influence of risk score and other clinical characteristics on poor prognosis. Finally, the nomogram was also used to assess the prognostic value.

### 2.5. Cell Culture

HOK (human oral epithelial cell line), HSC-3, HSC-4, and HSC-6 (human oral squamous cell line) were purchased from the Institute of Chinese Academy of Sciences (Shanghai, China). We cultured these cells in 1640 (Gibco, USA) medium with 10% fetal bovine serum (Gibco, USA) in an incubator, which was 37°C and 5% CO_2_ incubator.

### 2.6. RNA Extraction and RT-qPCR

Total RNA was extracted from HOK, HSC-3, HSC-4, and HSC-6 cell lines by using Trizol (Invitrogen). Subsequently, RNA concentration was measured by an ultraviolet spectrophotometer. We stored the extracted RNA samples at –80°C until use. The complementary deoxyribonucleic acids (cDNAs) were obtained by reverse transcription, and the SYBR Green method was used for PCR detection. GAPDH was used as internal references for mRNA, and the 2-*ΔΔ*Ct method was used to analyze the mRNA relative expression. The primer sequences of the ARGs were as follows: FADD: 5′-CGGCCTAGACCTCTTCTCCAT-3′ (forward), 5′-TGAGACTTTGAGCTGACGAGC-3′ (reverse); NKX2-3: 5′-GGAAGACGAGGGCGAGAAAT-3′ (forward), 5′-TCTAGAGACTTCTTCAGCTGGC-3′ (reverse).

### 2.7. HE and Immunohistochemical

We collected the tissue samples from the First Affiliated Hospital of Guangxi Medical University, which was approved by the Ethics Committee. All the paraffin-embedded sections were cut into 4 *μ*m sections. The HE and IHC staining for FADD proteins were then carried out in these tissue samples. We diluted the primary antibody FADD (Bioss, Beijing) with a ratio of 1 : 50. When finished, we observed the gene expression under the microscope. Finally, we took pictures of it and the magnifications used were 100x.

### 2.8. Statistical Analysis

The independent sample *t*-test and chi-squared test were used to compare the data of two groups. Statistical significance was defined as *p* < 0.05. All statistical analyses were carried out using the IBM SPSS software (version 25).

## 3. Results

### 3.1. Identification of Differentially Expressed ARGs between HNSCC Tissues and Adjacent Nontumor Tissues

**Figure 1:**
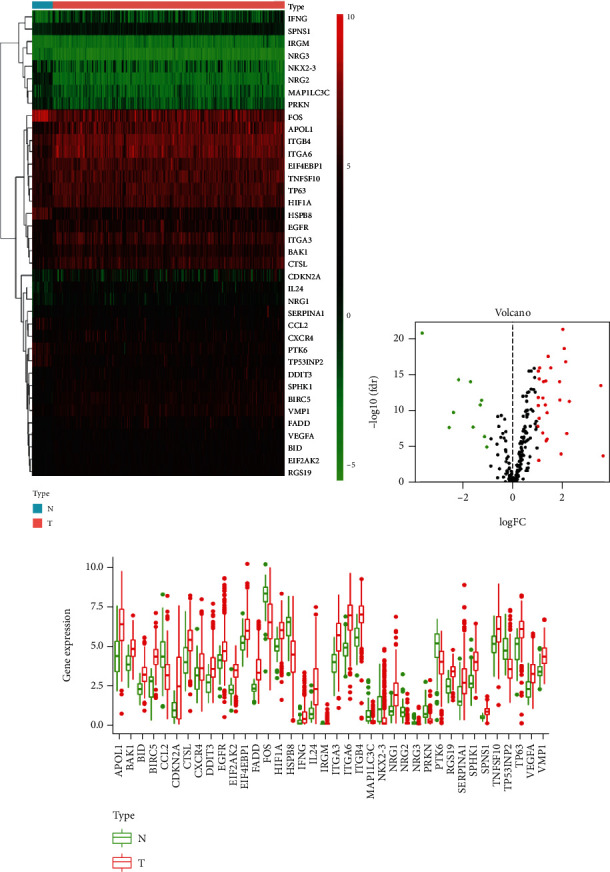
(a) Heat map and volcano plot showed the differentially expressed ARGs. (c) Box plot showed the differentially expressed ARG expression level between HNSCC tissues and nontumor tissues.

We involved a total of 502 primary HNSCC patients with gene expression data and clinical information in our study. At the same time, 232 ARGs derived from MSigDB were included in our study and there were 38 differentially expressed ARGs, including 28 upregulated (APOL1, BIRC5, VMP1, FADD, ITGB4, ITGA6, EIF4EBP1, TNFSF10, TP63, HIF1A, EGFR, ITGA3, BAK1, CTSL, CDKN2A, IL24, NGR1, SERPINA1, CXCR4, SPHK1, VEGFA, BID, EIF2AK2, RGS19, IFNG, DDIT3, SPNS1, and IRGM) and 10 downregulated ARGs (FOS, CCL2, HSPB8, PTK6, TP53INP2, NRG3, NKX2-3, NRG2, MAP1LC3C, and PRKN), with thresholds of ∣log2 fold change (FC) | >2. The heat map and volcano plot showed the differentially expressed ARGs, green represented low expression, and red represented high expression (Figures 1(a) and 1(b)). Finally, the box plot was generated. We applied it for visualizing the expression level of the differentially expressed ARGs between HNSCC tissues and nontumor tissues (Figure 1(c)).

### 3.2. GO and KEGG Enrichment Analysis of Differentially Expressed ARGs

In order to better understand the associated mechanisms in the development of HNSCC, we functionally categorized the differentially expressed ARGs. The Gene Ontology (GO) database denotes for numerous gene annotation terms, classified based on their association with biological processes (BP), molecule function (MF), and cellular component (CC). We then used the Kyoto Encyclopedia of Genes and Genomes (KEGG) to identify functional and pathways.

**Figure 2:**
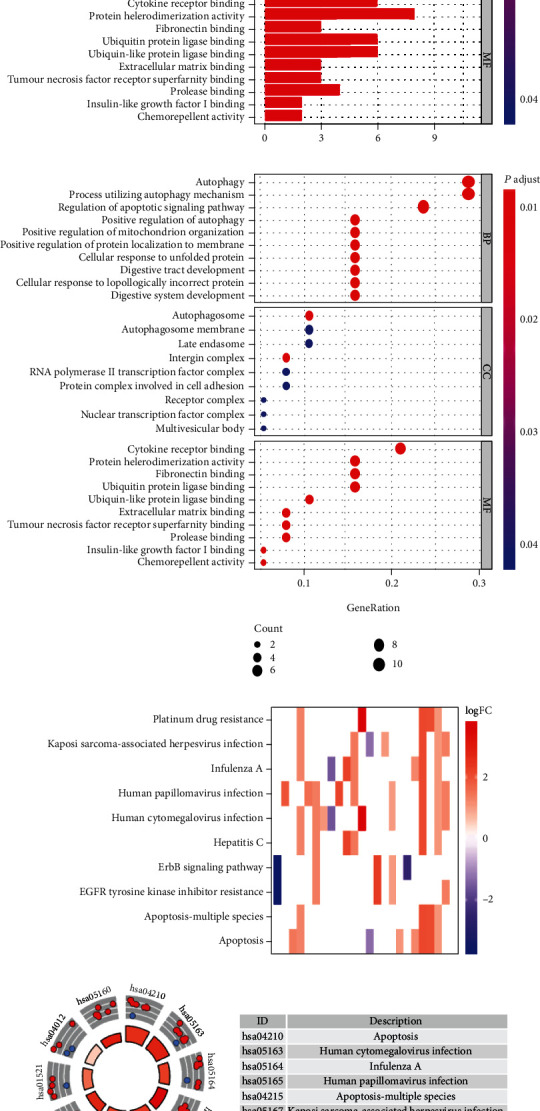
(a, b) Differentially expressed ARGs analyzed by GO enrichment. CC: cellular component; MF: molecular function; BP: biological process. (c, d) Enrichment of KEGG pathways of differentially expressed ARGs.

Figures 2(a) and 2(b) demonstrate the top 10 BP, MF, and CC, respectively, of GO enrichment, which is mainly involved in autophagy, positive regulation of mitochondrion organization, cellular response to topologically incorrect protein, positive regulation of protein localization to a membrane, nuclear transcription factor complex, and so on. Additionally, the top 10 of KEGG enrichment are summarized in Figures 2(c) and 2(d). KEGG enrichment shows that pathways of differentially expression ARGs mainly involve pathways in apoptosis, platinum drug resistance, EGFR tyrosine kinase inhibitor resistance, and ErbB signaling pathway.

### 3.3. Identification of Prognostic ARGs and Construction of the Prognosis Prediction Model

Differentially expressed ARGs, which were significantly associated with survival of patients, were screened to analyze ARGs involved in HNSCC progression. As a result, we identified two ARGs, including FADD and NKX2-3, and furthermore, the prognosis prediction model was constructed by these two prognostic ARGs. The prognosis prediction model = (0.138281∗FADD expression value) + (−0.449975∗NKX2‐3 expression value), and the calculated result was risk score.

**Figure 3:**
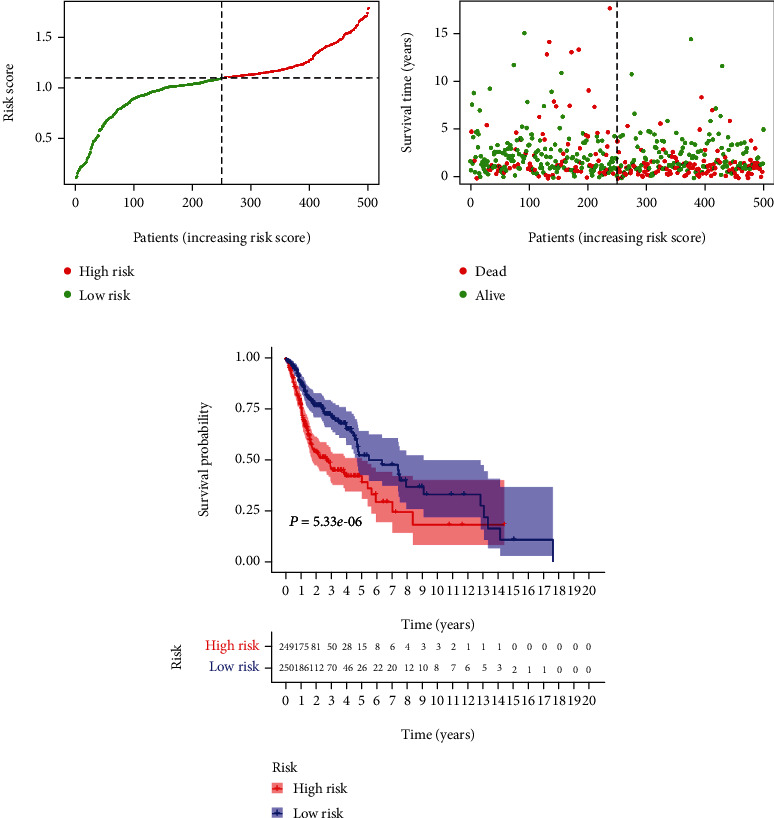
Construction of the prognosis prediction model.

Figures 3(a) and 3(b) demonstrate the distributions of risk score of HNSCC patients and the relationships between risk score and survival time. The abscissa represents the patients, and the risk score of the HNSCC cases in the TCGA database increases successively from left to right. The dotted line in the middle divides all the patients into the low-risk group and the high-risk group, which are shown in green and red, respectively. As can be seen from Figure 3(b), the survival time of patients decreases gradually from left to right, which means that the survival time of patients in the high-risk group is lower. To determine the role of the risk score in predicting the clinical prognosis of patients with HNSCC, a K-M survival curve was developed to analyze the differences in prognosis between the high-risk group and the low-risk group. K-M analysis showed that the survival rate was significantly lower in patients of the high-risk group than the low-risk group (Figure 3(c)).

### 3.4. Validation of the Risk Score Obtained by the Prognosis Prediction Model as an Independent Prognostic Indicator for HNSCC

**Figure 4:**
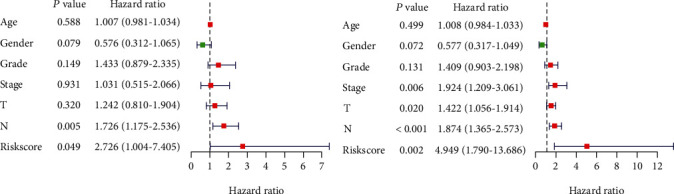
(a, b) Univariate and multivariate regression analysis of different clinicopathological variables (including the risk score) as independent prognostic indicators for HNSCC patients.

Univariate analysis showed that risk score was significantly related to the prognosis of patients (HR = 4.949; *p* = 0.002; Figure 4(a)). Then, after adjustment for other clinicopathologic features, multivariate analysis demonstrated that risk score remained an independent prognostic indicator for HNSCC patients (HR = 2.726; *p* = 0.049; Figure 4(b)). In addition, the *p* values of lymph node metastasis were also less than 0.05 in univariate analysis and multivariate analysis, which could be used as independent prognostic indicators as well. In the HNSCC data downloaded from TCGA, only 2 patients had distant metastasis, so we did not include metastasis as a factor in our analysis.

### 3.5. Construction of Nomogram

**Figure 5:**
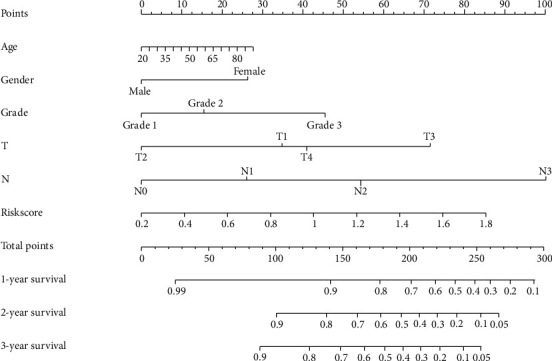
The nomogram was generated to predict the probability of 1-, 2-, and 3-year survival.

A nomogram always integrates multiple related factors, and each factor was assigned scores in proportion to its risk contribution to survival. By adding the total score of all these predicted factors to the total subscale, we were able to evaluate the prognosis of patients with HNSCC. It is a robust tool that has been used to quantitatively determine the risk of individuals in the clinical setting. In order to better apply risk score for clinical prediction, a nomogram was generated to predict the probability of 1-, 2-, and 3-year survival. As is shown in Figure 5, we involved the risk score, together with age, gender, tumor grade, tumor stage, tumor size, and lymph node metastasis into the nomogram.

### 3.6. The Relationships between Clinicopathological Parameters and Prognosis Prediction Model

**Figure 6:**
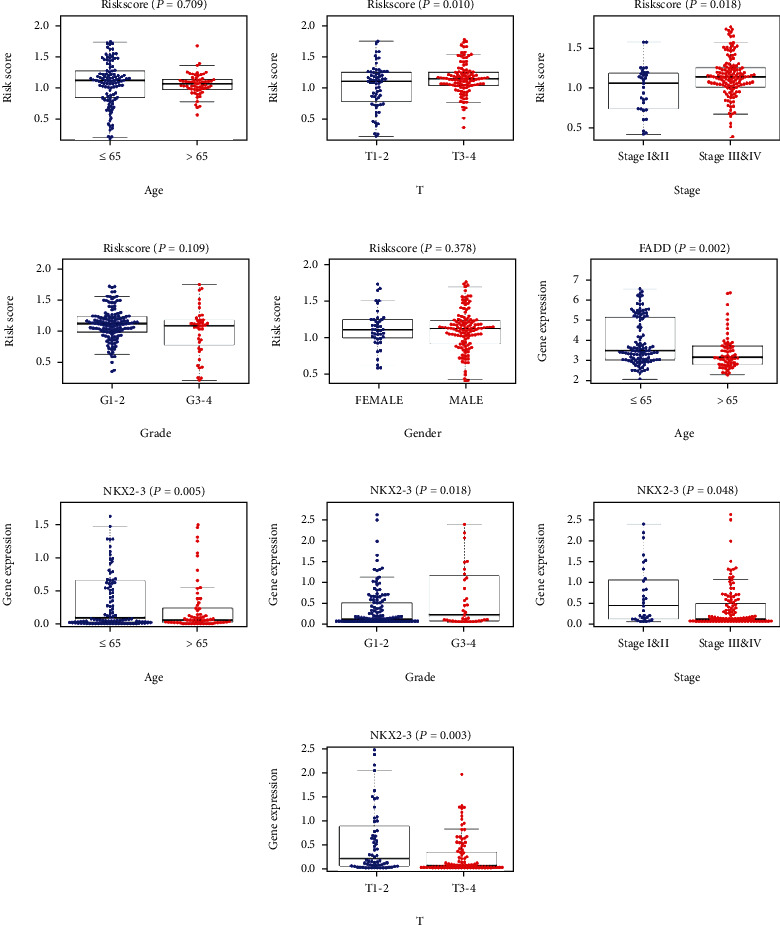
(a–e) Clinical correlation of risk score. (f) Clinical correlation of FADD. (g–j) Clinical correlation of NKX2-3.

The risk score of the prognosis prediction model was higher in T3-4 than in T1-2 (*p* = 0.01) and higher in Stage III&IV than in Stage I&II (*p* = 0.018). No difference of risk score was observed from age, gender, and grade (Figure 6). Similarly, we also calculated the correlation between prognosis-related ARGs and clinical characteristics. FADD was upregulated in patients younger than 65 years of age. Significant differences could also be observed in the expression of NKX2-3 in age, grade, stage, and tumor size (Figure 6).

### 3.7. Expression of FADD and NKX2 in Cell Lines and HNSCC Tissue Samples

**Figure 7:**
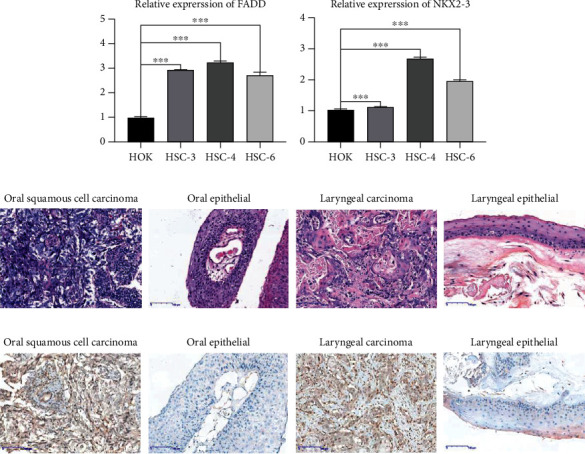
(a, b) RT-qPCR of FADD and NKX2-3 on HOK, HSC-3, HSC-4, and HSC-6 cells. (c, d) HE and IHC of FADD on HNSCC tissues and normal tissues.

We detected FADD and NKX2-3 expression in oral squamous cell carcinoma cell lines HSC-3, HSC-4, and HSC-6 and human oral squamous cell line HOK by RT-qPCR. Among them, the primary site of HSC-3 is the tongue squamous epithelium, while the primary site of HSC-4 and HSC-6 is the oral squamous epithelium. The results showed that the HSC-3, HSC-4, and HSC-6 cell lines all had a higher FADD expression level compared with that in HOK (Figure 7(a)). The results were all statistically different (*p* < 0.05), and the expression trend of FADD was consistent with the trend we analyzed in HNSCC (Figure 7(a)). On the contrary, we found that NKX2-3 had a higher expression level in the human oral squamous cell line HOK than HSC-3, which was consistent with the trend in our analysis. However, the expression levels of NKX2-3 in HSC-4 and HSC-6 were different (Figure 7(b)).

To further clarify the potential biological function of ARGs in HNSCC transformation, we next selected FADD, which performed well in cell lines, to characterize the expression changes in clinical samples by IHC. Tissues of oral squamous cell carcinoma and laryngeal carcinoma tissues were used for detection, both of which belong to HNSCC. We also collected normal oral epithelial tissues and laryngeal epithelial tissues as control groups for detection. We first performed HE staining to observe the pathological structure of these tissues (Figure 7(c)). For IHC, the identification of the staining was semiquantitative. Based on the results, score zero represented <5% positively stained cells, score one 6%-25% cells, score two 26-50%, and score three >50% [16]. For the final score, zero was considered negative, and 1-3 as positive. Positive expression of FADD proteins was detected in the oral squamous cell carcinoma and laryngeal carcinoma tissues (Figure 7(d)). Weak positive expression of FADD was seen in the oral epithelial tissues and laryngeal epithelial tissues (Figure 7(d)). The positive percentage of FADD was 65.2% (15/23) and 25.0% (2/8) in oral squamous cell carcinoma tissues and oral epithelial tissues, respectively (*p* < 0.05). Also, approximately 66.7% (14/21) of laryngeal carcinoma tissues and 28.6% (2/7) of laryngeal epithelial tissues were positive for FADD. The results were all statistically different (*p* < 0.05).

## 4. Discussion

In recent decades, the prognosis of HNSCC has been relatively poor, and treatment plans have been based primarily on the TNM stage. In addition, histopathological findings, such as marginal state, perineural infiltration, and lymphovascular invasion, are also used as the basis for treatment planning [17]. Previous studies have confirmed that autophagy always plays a crucial role in each stage of tumor [18, 19]. It could play an inhibitory role in the occurrence of cancer, and meanwhile, it could also promote cancer development. In addition, it is able to regulate the response of cancer cells to various therapies and treatments [20]. Thus, we believe that autophagy is associated with the development of HNSCC, and the exploration of autophagy mechanism opens up a new perspective for studying the new methods to the diagnosis and treatment of HNSCC [21].

In our study, we first obtained all ARGs from the public databases, then identified 38 differentially expressed ARGs in HNSCC samples, and finally selected 2 ARGs that related to the prognosis of patients with HNSCC. The prognosis prediction model was developed to predict the survival of HNSCC patients. The results demonstrated that the HNSCC patients with high-risk scores always resulted in poor overall survival. To better predict the survival of patients, we incorporated the risk score, age, gender, tumor grade, tumor stage, tumor size, and lymph node metastasis to construct a nomogram. Our results showed that the risk score has a high prognostic value and it could be used as a diagnostic biomarker. Finally, we analyzed the clinical features of two prognostic ARGs that we used to construct the prognostic model and calculate the risk score. According to our analysis and experimental results, these two genes have significant differences both in cell lines and clinical samples, which means they are closely associated with the occurrence and development of HNSCC. They are able to be used as therapeutic targets for HNSCC. Among these two ARGs, Fas-associated protein with death domain is the full name for FADD. FADD is a key adaptor protein that mediates apoptosis signal transduction [22]. Excepted for being related to apoptosis, FADD is also involved in the process of cell cycle progression, proliferation, innate immunity, tumor development, inflammation, and autophagy [23]. Therefore, during many essential cellular processes, FADD is a significant and special controller [24]. This gene is believed to be the driver gene for chromosomal region 11q13.3 amplification [25]. FADD often has a high expression trend in HNSCC. Also, a study found that high FADD expression always results in worse OS, DSS, and DFS in patients with HNSCC and thus has potential values for prognostic prediction and treatment planning development [26, 27]. A meta-analysis has found that immunohistochemical assessment of FADD could be incorporated into the prognostic evaluation of HNSCC [28]. In addition, NKX2-3 is a member of the NKX family, which is a series of the homeodomain transcription factors. NKX2-3 plays crucial roles in many biologic processes, including cell proliferation and growth, metabolic process, immune and inflammatory response, and angiogenesis [29, 30]. However, the abnormal expression of NKX2-3 is mostly seen in digestive system diseases, which has been considered as a gene related to inflammatory bowel disease and Crohn's disease [31, 32]. A study has found that NKX2-3 could regulate the Wnt signaling pathway and then play a critical role in the pathogenesis of colorectal cancer and could be a new biomarker for clinical practice, including early diagnosis and subsequent therapy [33, 34].

In this study, a prognosis prediction for HNSCC was established and it showed good predictive ability, which provided useful guidance for the prediction of individual patients. In addition, we confirmed the critical role of ARGs, including NKX2-3 and FADD, during the occurrence and development of HNSCC for the first time, which can be used as a diagnostic biomarker and a therapeutic target for HNSCC. However, there are still some limitations in our study. This is a study based on bioinformatics analysis, and the data were acquired from the public database. Thus, we need more independent cohorts to prove the effectiveness of the model we constructed. Additionally, although we did some preliminary validation on the 2 ARGs, their mechanism needs further functional experimental research and more clinical samples to verify. In conclusion, this study provides new strategies for the diagnosis, prognosis prediction, and treatment of patients with HNSCC.

## Data Availability

All data included in this study are available from the corresponding author upon request.

## References

[1] Argiris A., Karamouzis M. V., Raben D., Ferris R. L. (2008). Head and neck cancer. *The Lancet*.

[2] Jou A., Hess J. (2017). Epidemiology and molecular biology of head and neck cancer. *Oncology Research and Treatment*.

[3] Arantes L., de Carvalho A. C., Melendez M. E., Lopes Carvalho A. (2018). Serum, plasma and saliva biomarkers for head and neck cancer. *Expert Review of Molecular Diagnostics*.

[4] Budach V., Tinhofer I. (2019). Novel prognostic clinical factors and biomarkers for outcome prediction in head and neck cancer: a systematic review. *The Lancet Oncology*.

[5] Glastonbury C. M. (2020). Head and neck squamous cell cancer: approach to staging and surveillance. *Diseases of the Brain, Head and Neck, Spine 2020–2023. IDKD Springer Series*.

[6] Economopoulou P., de Bree R., Kotsantis I., Psyrri A. (2019). Diagnostic tumor markers in head and neck squamous cell carcinoma (HNSCC) in the clinical setting. *Frontiers in Oncology*.

[7] Lyamzaev K. G., Tokarchuk A. V., Panteleeva A. A., Mulkidjanian A. Y., Skulachev V. P., Chernyak B. V. (2018). Induction of autophagy by depolarization of mitochondria. *Autophagy*.

[8] Hernández-Tiedra S., Fabriàs G., Dávila D. (2016). Dihydroceramide accumulation mediates cytotoxic autophagy of cancer cells via autolysosome destabilization. *Autophagy*.

[9] Boya P., Reggiori F., Codogno P. (2013). Emerging regulation and functions of autophagy. *Nature Cell Biology*.

[10] Tektemur A., Ozaydin S., Etem Onalan E. (2019). TRPM2 mediates distruption of autophagy machinery and correlates with the grade level in prostate cancer. *Journal of Cancer Research and Clinical Oncology*.

[11] Yao Z. Q., Zhang X., Zhen Y. (2018). A novel small-molecule activator of sirtuin-1 induces autophagic cell death/mitophagy as a potential therapeutic strategy in glioblastoma. *Cell Death & Disease*.

[12] The Cancer Genome Atlas Research Network, Weinstein J. N., Collisson E. A. (2013). The Cancer Genome Atlas pan-cancer analysis project. *Nature Genetics*.

[13] Liberzon A., Birger C., Thorvaldsdóttir H., Ghandi M., Mesirov J. P., Tamayo P. (2015). The Molecular Signatures Database Hallmark Gene Set Collection. *Cell Systems*.

[14] Ritchie M. E., Phipson B., Wu D. (2015). limma powers differential expression analyses for RNA-sequencing and microarray studies. *Nucleic Acids Research*.

[15] Yu G., Wang L. G., Han Y., He Q. Y. (2012). clusterProfiler: an R package for comparing biological themes among gene clusters. *OMICS*.

[16] Zhu L., Yan D., Chen Y., Chen S., Chen N., Han J. (2020). The identification of autophagy-related genes in the prognosis of oral squamous cell carcinoma. *Oral Diseases*.

[17] Liu C., Yu Z., Huang S. (2019). Combined identification of three miRNAs in serum as effective diagnostic biomarkers for HNSCC. *eBioMedicine*.

[18] Onorati A. V., Dyczynski M., Ojha R., Amaravadi R. K. (2018). Targeting autophagy in cancer. *Cancer*.

[19] Scrivo A., Bourdenx M., Pampliega O., Cuervo A. M. (2018). Selective autophagy as a potential therapeutic target for neurodegenerative disorders. *Lancet Neurology*.

[20] Mizushima N., Levine B. (2020). Autophagy in human diseases. *The New England Journal of Medicine*.

[21] Glick D., Barth S., Macleod K. F. (2010). Autophagy: cellular and molecular mechanisms. *The Journal of Pathology*.

[22] Mouasni S., Tourneur L. (2018). FADD at the crossroads between cancer and inflammation. *Trends in Immunology*.

[23] Schwarzer R., Jiao H., Wachsmuth L., Tresch A., Pasparakis M. (2020). FADD and caspase-8 regulate gut homeostasis and inflammation by controlling MLKL- and GSDMD-mediated death of intestinal epithelial cells. *Immunity*.

[24] Tourneur L., Chiocchia G. (2010). FADD: a regulator of life and death. *Trends in Immunology*.

[25] Pattje W. J., Melchers L. J., Slagter-Menkema L. (2013). FADD expression is associated with regional and distant metastasis in squamous cell carcinoma of the head and neck. *Histopathology*.

[26] Rasamny J. J., Allak A., Krook K. A. (2012). Cyclin D1 and FADD as biomarkers in head and neck squamous cell carcinoma. *Otolaryngology and Head and Neck Surgery*.

[27] Marín-Rubio J. L., Vela-Martín L., Fernández-Piqueras J., Villa-Morales M. (2019). FADD in cancer: mechanisms of altered expression and function, and clinical implications. *Cancers*.

[28] González-Moles M. Á., Ayén Á., González-Ruiz I. (2020). Prognostic and clinicopathological significance of FADD upregulation in head and neck squamous cell carcinoma: a systematic review and meta-analysis. *Cancers*.

[29] Yu W., Hegarty J. P., Berg A. (2011). NKX2-3 transcriptional regulation of endothelin-1 and VEGF signaling in human intestinal microvascular endothelial cells. *PLoS One*.

[30] Kerkhofs C., Stevens S., Faust S. N. (2020). Mutations in RPSA and NKX2-3 link development of the spleen and intestinal vasculature. *Human Mutation*.

[31] Yu W., Lin Z., Kelly A. A. (2009). Association of a Nkx2-3 polymorphism with Crohn’s disease and expression of Nkx2-3 is up-regulated in B cell lines and intestinal tissues with Crohn’s disease. *Journal of Crohn's & Colitis*.

[32] Vojkovics D., Kellermayer Z., Gábris F. (2019). Differential effects of the absence of Nkx2-3 and MAdCAM-1 on the distribution of intestinal type 3 innate lymphoid cells and postnatal SILT formation in mice. *Frontiers in Immunology*.

[33] Yu W., Lin Z., Pastor D. M. (2010). Genes regulated by Nkx2-3 in sporadic and inflammatory bowel disease-associated colorectal cancer cell lines. *Digestive Diseases and Sciences*.

[34] Vojkovics D., Kellermayer Z., Kajtár B., Roncador G., Vincze Á., Balogh P. (2018). Nkx2-3-a slippery slope from development through inflammation toward hematopoietic malignancies. *Biomarker Insights*.

